# WILL (When to Induce Labour to Limit risk in pregnancy hypertension): a multicentre randomised controlled trial — adaptations to deliver a timing-of-birth trial during the COVID-19 international pandemic

**DOI:** 10.1186/s13063-022-06834-4

**Published:** 2022-10-21

**Authors:** Laura A. Magee, Sue Tohill, Katie Kirkham, Ruth Evans, Eleni Gkini, Catherine A. Moakes, Clive Stubbs, Jim Thornton, Peter von Dadelszen, the WILL Trial Study Group, Peter Brocklehurst, Lucy Chappell, Jon Dorling, Marcus Green, Pollyanna Hardy, Jennifer Hutcheon, Katie Kirkham, Catherine Moakes, Ben Mol, Katie Morris, Paul Riley, Tracy Roberts, Janet Scott, Joel Singer, Ruth Unstead-Joss, Julie Wade, Ben W. Mol, Tim Draycott, Graeme MacLennan, Lucy MacKillop, Paul Mannix, Diana Elbourne, Henk Groen, Edile Murdoch, Sarah Stock, Sumita Bhuiya, Soumendra Nallapeta, Emma Dooks, Sophie Packham, Diane Whitehouse, Chloe O’Hara, Connie Weston, Diane Mellers, Lesley Brittain, Phern Adams, Rebecca Shakespeare, Sudeepthi Kakara, Janet Wright, Amal Mighell, Jennifer Syson, Kari Swettenham, Jenny Eedle, Shaila Seraj, Maryanne Bray, Bethan Jones, Claire Bertorelli, Hannah Ritter, Vikki Keeping, Janet Cresswell, Mary Kelly-Baxter, Li-Shan Yeoh, Shailly Sahu Bhansali, Vandana More, Bini Ajay, Geraldine Upson, Danielle Hake, Diana Opoku, Emma Wayman, Natalia Cwiek, Stacy Tregellas, Nikki Lee, Lavinia Margarit, Joelle Pike, Kate Jones, Sophie-Mae Wheeler-Davies, Meena Ali, Indhuja Rajkumar, Ruth Habibi, Sarah Davies, Anangsha Kumar, Harinakshi Salian, Trudy Smith, Deepika Meneni, Hazel Alexander, Helen Harwood, Kerry Hebbron, Lynn Whitecross, Mary Hodgers, Shilpa Mahadasu, Nick Kametas, Yasmin Sana, Hayley Martin, Rebecca Jarman, Sophie Webster, Jyothi Rajeswary, Mandy Gill, Gabrielle Bambridge, Isabel Bradley, Kristina Sexton, Lola Oshodi, Cornelia Wiesender, Claire Dodd, Rupa Modi, Beverley Cowlishaw, Gina Mulheron, Magdalena Kierzenkowska, Molly Patterson, Patricia Amos, Sharon Marie Bates, Sharon Raper, Umber Agarwal, Ruth Cockerill, Amy Mahdi, Caroline Cunningham, Michelle Dower, Sian Andrews, Siobhan Holt, Carly Williams, Zora Castling, Linda Watkins, David Churchill, Ellmina McKenzie, Julie Icke, Laura Devison, Vinita Raheja, Angela Ayuk, Jessica Reynolds, Julie Wyton, Stacey Duffy, Kate Walker, Jane Cantliffe, Catriona Hussain, Carys Smith, Harriet Anderson, Lesley Hodgen, Karen Brackley, Nicki Martin, Fiona Walbridge, Rhea Hampton, Nia Jones, Patrick Bose, Catherine Young, Fidelma Lee, Rebecca Peart, Emma Tanton, Kat Rhead, Kristin Fiedler, Ruth Bowen, Stephy Mathen, Zainab Sarwar, Chloe Rishton, Chloe Scott, Jane Farey, Nisha Verasingam, Mel Rich, Annette Moreton, Catherine Bressington, Jennifer Pullen, Sara Burnard, Wendy Duberry, Madhuchanda Dey, Sharon Jones, Pauline Bird, Aarti Ullal, Eileen Walton, Ashleigh Price, Janet Scollen, Judith Ormonde, Kirsten Herdman, Lesley Hewitt, Lucy Rowland, Mandeep Singh, Sundararajah Raajkumar, Beena Saji, Asma Khalil, Alice Perry, Emily Marler, Ijeoma Imuzeze, Sophie Robinson, Jonathan Nelson, Kathryn McNamara, Carina Craig, Del Endersby, Jayne Wagstaff, Kate Robinson, Hannah Barnes, Jane Gavin, Jenny Myers, Kate Stanbury, Christine Hughes, Latha Vinayakarao, Louise Melson, Stephanie Grigsby, Susara Blunden, Melanie Griffin, Sarah Newell, Katharine Jane Thompson, Brittany Smart, Elizabeth Payne, Marie Pitchford, Rahila Khan, Sophia Stone, Ahmed Elgarhy, Emma Meadows, Marian Flynn-Batham, Nikky Passmore, Vivienne Cannons, Declan Symington, Alice Lewin, Hayley Tarft, Jessamine Hunt, Zoe Vowles, Maria Slaney, Rachel Woodcock, Alex Van der Meer, Tracey Benn, Ru Davies, Sophie Boyd, Gareth Waring, Jill Riches, Andrea Fenn, Aly Kimber, Susan Harrop, Daniel Stott, Amos Tetteh, Davide Casagrandi, Miriam Bourke, Eirini Vaikousi, Rita Sarquis, Morenike Folorunsho, Olivia Newth, Sarah Weist, Yaa Acheampong, Vidhya Ravikumar, Jemma Yorke, Vicki Atkinson, Shelly Wood, Tigist Mengistu, Robert Chadwick, Helen Haden, Lisa Richardson, Joanna Girling, Amy Barker, Andrea Day, Elaine Palmer, Louise Page, Millicent Nwandison, Osaeloke Osakwe, Philippe de Rosnay, Sana Usman, Susan Barnes, Grace Ryan, Komal Lal, Lauren Trepte, Samantha Steele, Jacqueline Tang, Harriet Pearson, Jo Ingham, Nicola Spark

**Affiliations:** 1 Institute of Women and Children’s Health, King’s College London, 5thFloor, Addison House, Guy’s Campus, London, SE1 1UL UK; 2 Department of Women and Children’s Health, School of Life Course Sciences, Faculty of Life Sciences and Medicine, King’s College London, London, UK; 3 Maternity Services, Guy’s and St Thomas’ NHS Foundation Trust, London, UK; 4 Birmingham Clinical Trials Unit, University of Birmingham, Birmingham, UK; 5 Department of Obstetrics and Gynaecology, University of Nottingham, Nottingham, UK

**Keywords:** Trial management, Remote consent, COVID, Pregnancy, Timing of birth

## Abstract

**Background:**

As a pragmatic randomised timing-of-birth trial, WILL adapted its trial procedures in response to the COVID-19 pandemic. These are reviewed here to inform post-pandemic trial methodology.

**Methods:**

The trial (internal pilot) paused in March 2020, re-opened in July 2020, and is currently recruiting in 37 UK NHS consultant-led maternity units. We evaluated pandemic adaptations made to WILL processes and surveyed sites for their views of these changes (20 sites, videoconference).

**Results:**

Despite 88% of sites favouring an electronic investigator site file (ISF), information technology requirements and clinical trial unit (CTU) operating procedures mandated the ongoing use of paper ISFs; site start-up delays resulted from restricted access to the CTU. Site initiation visits (SIVs) were conducted remotely; 50% of sites preferred remote SIVs and 44% felt that it was trial-dependent, while few preferred SIVs in-person as standard procedure. The Central team felt remote SIVs provided scheduling and attendance flexibility (for sites and trial staff), the option of recording discussions for missing or future staff, improved efficiency by having multiple sites attend, and time and cost savings; the negative impact on rapport-building and interaction was partially mitigated over time with more familiarity with technology and new ways-of-working. Two methods of remote consent were developed and used by 30/37 sites and for 54/156 recruits. Most (86%) sites using remote consenting felt it improved recruitment. For remote data monitoring (5 sites), advantages were primarily for the monitor (e.g. flexibility, no time constraints, reduced cost), and disadvantages primarily for the sites (e.g. document and access preparation, attendance at a follow-up meeting), but 81% of sites desired having the option of remote monitoring post-pandemic.

**Conclusions:**

COVID adaptations to WILL trial processes improved the flexibility of trial delivery, for Central and site staff, and participants. Flexibility to use these strategies should be retained post-pandemic.

**Trial registration:**

ISRCTN77258279. Registered on 05 December 2018.

## Background

The worldwide SARS-CoV-2 pandemic has changed the way that we conduct healthcare research. In response, many investigators have made ‘COVID adaptations’ that have allowed scientific inquiry to continue into the questions that we must ask and answer to improve healthcare outcomes both related and unrelated to COVID-19.

In September 2018 in the UK, the Medicines and Healthcare products Regulatory Agency (MHRA) [1] and Health Research Authority (HRA) [2] released a joint statement that set out the legal and ethical requirements in the UK for seeking and documenting consent for research, using electronic methods [3]. As such, pre-pandemic, many procedures for remote trial delivery were in use, or approved for use, but many were not or had not been adapted for routine use within clinical trial delivery, due predominantly to the standard operating procedures adopted by sponsors and clinical trial units (CTUs), to ensure their compliance with good clinical practice (GCP).

It is useful to reflect on which of the many adjustments to trial procedures that were made or expanded in use during the pandemic worked well and which have not, to best inform our post-pandemic (‘endemic’) methods. These include, but are not limited to, remote and electronic consent used pre-pandemic to overcome barriers to recruitment, such as remote access (addressed by videoconferencing and then ‘teleconsent’ [4]), or the need for timely intervention (such as in acute stroke when the legal representative is not physically present in hospital with the patient [5]).

The WILL trial (When to Induce Labour to Limit risk in pregnancy hypertension) is a United Kingdom (UK) multicentre, NIHR-funded, randomised controlled trial of timing-of-birth for women with chronic or gestational hypertension. We describe our COVID pandemic adaptations to inform post-pandemic methodology.

## Methods

The WILL trial aims to investigate the clinical effectiveness and cost-consequences of planned early-term delivery at 38 ^+ 0^ to 38 ^+ 3^ weeks’ gestation, compared with expectant care at term until at least 40 ^+ 0^ weeks’ gestation, in pregnant women with chronic or gestational hypertension that develops by 37 ^+ 6^ weeks’ gestation. In brief, the design is a pragmatic, parallel-group, open-label, multicentre, randomised controlled trial (with an internal pilot). Included are women aged ≥ 16 years, with a diagnosis of chronic or gestational hypertension, singleton pregnancy, live fetus, gestational age of 36 ^+ 0^ to 37 ^+ 6^ weeks, and able to give documented informed consent to participate; excluded are women with a contraindication to either one of the trial arms (e.g. evidence of pre-eclampsia), blood pressure (BP) ≥ 160 mmHg systolic or ≥ 110 mmHg diastolic until < 160/110 mmHg, a major fetal anomaly anticipated to require neonatal unit admission, or participation in another timing of the delivery trial. The co-primary outcomes are maternal (composite of poor maternal outcome until primary hospital discharge home or 28 days after birth (whichever is earlier), defined as severe hypertension, death, or morbidity) and neonatal (neonatal care unit admission for ≥ 4 h, until primary hospital discharge home or 28 days after birth, whichever is earlier). The key secondary outcome is Caesarean birth. The trial will recruit 1080 pregnant women with chronic or gestational hypertension, of which just over 20% have been recruited to date, from NHS consultant-led maternity units (for further details, see https://www.birmingham.ac.uk/research/bctu/trials/womens/will/will.aspx).

The trial was paused on 20 March 2020, at the end of the internal pilot trial, when the first wave of the pandemic began. WILL began its main phase on 9 July 2020 and, since then, has remained open to recruitment throughout subsequent COVID-19 waves. Throughout England and Wales, we have opened 23 new main trial phase sites and re-opened 18 pilot sites (of which one has now closed and one is paused), consented 265 women, and randomised 246 as of 26 November 2021. WILL has just exceeded its pre-COVID monthly rate of randomisation [https://www.birmingham.ac.uk/research/bctu/trials/womens/WILL/WILL.aspx].

In this paper, we present a descriptive analysis of how WILL processes, including site start-up, recruitment, data monitoring, and other active site activities, were adapted during the COVID pandemic. These processes have not been formally evaluated. Also, we present the results of a site ‘slido’ survey undertaken as a polling option during our 16 November 2021 monthly site Zoom videoconference at which each site is represented by one individual, or two or more persons join on the same Zoom link.

## Results

### Site start-up

Changes impacting site start-up have been seen in three primary areas: (i) staff redeployment to clinical care and COVID-19 research, with the effect that previous interest in WILL participation was not as strong; (ii) the nature of the investigator site file (ISF) and trial management file (TMF); and (iii) remote retraining and site initiation visits (SIVs).

Of the 18 internal pilot phase sites that re-opened, many reported struggling with enough staff time to support the trial, and some (*N* = 8) re-paused for recruitment at some point. We have had 15 sites withdraw their pre-pandemic written expressions of interest due to staff redeployment or loss, with an additional 16 sites deferring decisions until 2022.

We explored converting the ISF (and TMF) from paper to an electronic format, having considered potential advantages and disadvantages, the enthusiasm from our sites, and the fact that most of the ISF is rarely if ever used by sites (e.g. correspondence about submission and approval of amendments) (Table 1). The Birmingham Clinical Trial Unit’s (BCTU’s) online file-share server, BEAR DataShare (the BCTU’s peer-to-peer, cloud-based file-sharing application), did not have all of the capabilities necessary to manage an electronic TMF in compliance with the European Medicines Evaluation Agency guidance (2018). Ultimately, the significant information technology (IT) resource implications (including secure access, appropriate file storage, document change management, and version control) and costs made this unfeasible during the pandemic. As such, the requirement to have paper files in the event of an inspection led to continued use of the paper ISF, in line with the BCTU’s current standard opening procedures. Nevertheless, an online repository was established to facilitate ease of access for ISF documents, with the intention of printing and filing within the ISF at sites; while not a fully electronic ISF, this approach gave sites ease of access to trial documentation.

**Table 1:** Advantages and disadvantages of a paper vs. electronic ISF

Paper ISF		Electronic ISF	
**Advantages**	**Disadvantages**	**Advantages**	**Disadvantages**
Visible and easy to store on shelves	Only available to site staff working where ISF is stored	Accessible by all site staff with an electronic device connected to a secure network	Requires a separate storage system
Easier to browse and check if something is missing	Undergoes wear and tear and becomes more cumbersome to use	Does not degrade or take up more physical space as documents are added	Identifying the ISF is more reliant on the clear naming of the document
Easy to update	-	-	Superseding documents require documents to be moved from the ‘Current’ folder to the ‘Superseded’ folder, and the ‘Superseded’ label must be applied to the document
Stored together with other key trial paperwork	As documents accumulate, binders fill up and new ones are required, and there is a risk that they will be separated	Can store all ISF documents together, including ICFs and trial stationery	Sites can update their folders by downloading new documents from the trial website/platform, although with an advanced eTMF system, sites would receive them automatically and sign-off [e.g. for new protocol] is done electronically, without printing
Low tech and available to all	-	Once the eISF system is set up, printing costs are reduced	Substantial IT set-up costs

*eISF* Electronic ISF,* ISF* Investigator site file,* TMF* Trial management file

Preparing the paper ISF during the lockdown and remote working from home was problematic for the Central team. During the lockdown, approvals were required to attend the CTU on-site to make up the binders and ship them. However, as BEAR DataShare could offer sites secure access to files, we provided site staff with an electronic repository of ISF documents for ease of working at a distance from the ISF binder, whether in the Trust or from home.

When WILL restarted on 9 July 2020, we moved directly to remotely conducting refresher training for pilot sites, and SIVs for new sites, to ensure that the site investigator and study staff understood the protocol and trial operational steps. While formerly offered as face-to-face visits, we moved to offering these remotely, by Zoom or Teams, depending on the Trust. We conducted four retraining sessions (three with multiple sites) and 16 new SIVs (six with multiple sites).

The Central team identified a number of advantages of remote retraining and SIVs. If an important site team member could not attend a date that worked for most others, or if there was a sudden change of plans regarding attendance near to the meeting, some of the training could be achieved and with the permission of the team, a recording provided to those team members who were absent, as well as for future team members. If the site PI could not make it or if additional discussion was desired, the WILL CI made a follow-up call to the PI. Often, the full complement of five Central team members were present at the SIV (in contrast to prior selective attendance), including the CI, senior trial manager, senior data manager, and one or both of our lead research midwives. The WILL research midwife routinely followed up with the site’s lead research midwife by phone or video link.

We estimated that remote SIVs (vs. prior face-to-face meetings) saved an average of 6 h of travel time per meeting, with an estimated travel cost savings of approximately £250 per meeting, based on two team members attending.

There were, however, some obvious disadvantages of remote training, at least for some sites and some meetings. Some were technical and decreased in frequency over time, such as unstable internet, difficulties using the video camera, not muting to avoid interference from background noise in the room, and speaking over others by not raising one’s hand electronically (or on camera). Central team members were unclear at times whether staff who turned off their cameras were engaged. With multiple sites in attendance, it was sometimes difficult to problem-solve for individual sites in set-up. Other challenges included a perceived reduction in questions (vs. at a face-to-face meeting), at least at some site visits. There was a general sense that it was more difficult to build a rapport, especially if many staff and/or sites were on the call. Database training was less interactive, as it was harder to follow on screen.

### Recruitment and active site activities

The WILL team developed a process for remote consent in July 2020, as discussed below. Randomisation was always a remote process, based on standardised questions designed to ensure that women had not developed a contraindication to randomisation since consent, usually taken within the prior week. These questions were aimed at identifying any new plans for delivery and detecting potential progression to pre-eclampsia that would warrant re-evaluation by the clinical care team.

**Fig. 1:**
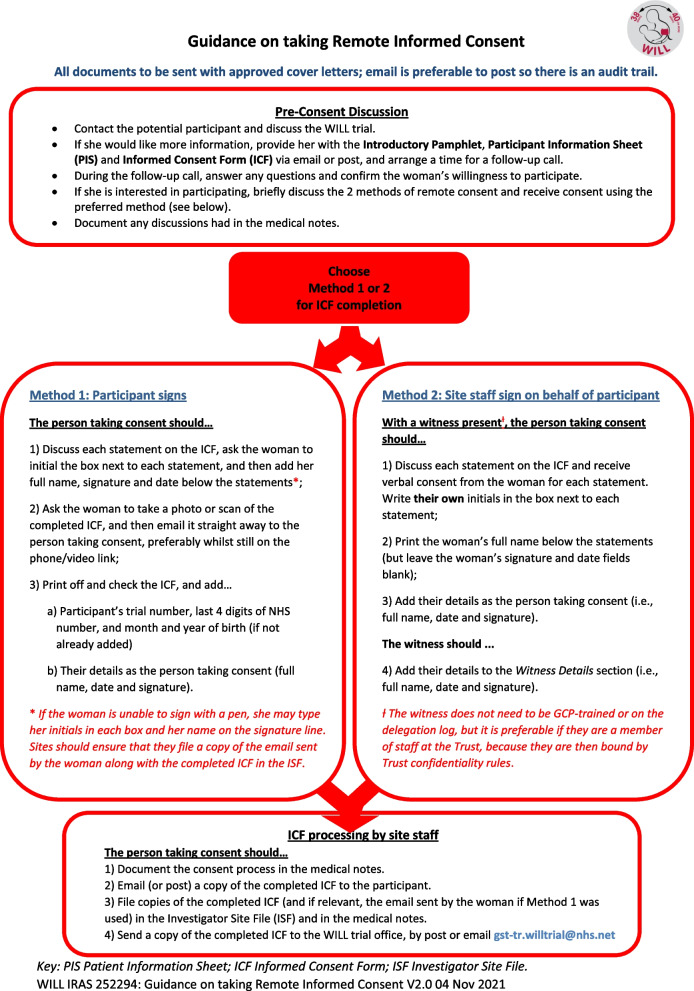
Two novel ‘low tech’ approaches to remote consent

The WILL trial team developed two novel ‘low tech’ approaches to remote consent (Fig. 1). In ‘Method 1’, the participant signs a paper copy (received directly or printed from an email) and sends it back (as the paper copy or as a scan) to the site staff. In ‘Method 2’, the participant gives verbal informed consent, the site staff sign, and another site staff member then counter-signs, acting as a witness; then, the site staff send a copy of the consent form to the woman for her records. Each method relies on local methods of remote identification and communication strategies (i.e. telephone or video link) approved for clinical care.

Remote consent was approved within 16 days by the trial sponsor (Guy’s and St. Thomas’ NHS Foundation Trust), as a rapid COVID adaptation that did not require research ethics committee approval prior to implementation; however, remote consent was included in our next ethics amendment. Of the 37 sites open to recruitment from that point or at some point thereafter, 30 (81%) have used a remote consent method. The process was first used on the 3rd of August 2020, and since then, 54/156 women who have joined the trial have consented remotely — 19 using ‘Method 1’ and 35 using ‘Method 2’. No concerns have been expressed by sites about security measures, such as a lack of encryption.

Active sites are invited to a monthly operational meeting, which was held by telephone pre-COVID, and is now held by Zoom videoconference. These have been well attended, with consistently more than 25 participating sites, whether by video or telephone link, and not infrequently, with clinical and research staff logging on from home on days off. Active site participation has been enhanced by the use of SLiDO questionnaires to poll opinions about important operational issues, such as languages for translation of study materials, barriers to recruitment, or quizzes about pregnancy hypertension and WILL protocol facts.

Also, the chief investigator (LAM) has been able to accommodate all requests for talks at trial site clinical governance meetings, held for maternity care clinicians and research staff, to discuss clinical, operational, and research issues. These talks were previously challenging to organise, given travel to and from site locations and the fact that most trusts hold these meetings on Friday afternoon and only monthly.

### Data monitoring

The WILL Data Monitoring Plan stipulates that monitoring will be conducted by the WILL Central team for five pilot sites with at least five participants each and will be extended to further recruiting sites if issues are identified that relate to trial conduct, data quality, or patient safety (reporting). Since March 2020, the Central team pivoted to a remote process, rather than defer on-site monitoring until after the pandemic. The Central team sought advice from the sites to ascertain the most pragmatic methods for conducting data monitoring and a number of methods were suggested: (i) scanning and forwarding redacted notes to the monitor via electronic means (i.e. NHS secure email or ‘EDGE’, a cloud-based clinical research management system) and (ii) granting monitor access to electronic patient records (although this method has yet to be used as there are data protection issues related to third-party personnel accessing patient identifiable data from outside the trust).

Five sites have undergone their data monitoring remotely, all by the same Central team members. These sites responded to a survey about their experiences in (i) preparing for data monitoring (i.e. which source documents would be needed, accessing and printing source documents required, sharing source documents with the monitor); (ii) having a follow-up meeting to review the monitoring report; and (iii) a comparison of remote (vs. in-person, on-site) visits with regard to their preferences, resource implications (staffing, rooms, access, and equipment), and the amount of site time required. The feedback is summarised descriptively in Table 2. It appeared that the advantages were primarily for the Central team, and although it was advantageous for the site not to have to book physical space and time, the site teams reported greater resource implications, and a spectrum of opinion from negative to very positive.

**Table 2:** Advantages and disadvantages of remote (vs. face-to-face, on-site) data monitoring

	**Advantages**	**Disadvantages**
**Preparing for monitoring**	Convenient, visits easy to schedule, resource-light at sites (as no need for room bookings or equipment), and inexpensive for the Central team (with no travel or accommodation costs)	Loss of direct contact between sites and trial centre
	Less time-consuming for the monitor as no travel time	May be more time-consuming and resource-intensive for the site, given printing and redacting/anonymising source documents, but there was no agreement about this
	Sites can share documents in numerous ways, such as by the Cloud, NHS email, EDGE software, hard copy in the post, or screen sharing	Monitor must be specific about what source data they want to view, other important notes may not be shared with the monitor unless they have been requested. May be more difficult to resolve queries ‘on the spot’. If additional documents are requested ‘ad hoc’, there may be a delay before the monitor receives them
	No scheduling restrictions for the monitor — the visit can take place at the convenience of the monitor	Extra considerations for data protection — especially if source documents are being monitored outside of the workplace (e.g. working from home)
		Monitor must agree to destroy/delete source data immediately after the monitoring visit is completed
		Monitor cannot remotely monitor the paper ISF, but must rely on sites to complete an ISF checklist in order to verify the completeness of the ISF
**Follow up meeting**	No restriction on the number of people invited	A separate meeting must be scheduled to discuss the monitoring report
**Other feedback**	Sites expressed a spectrum of opinion about remote monitoring, with regard to the preference for, fewer resource implications (human and research), and less time required to undertake (each varying from ‘disagree’ to ‘strongly agree’)	

Changes were made to the trial’s risk assessment, to incorporate the processes of remote consent and data monitoring. Further details were provided on the verification of patient identity and a recommendation to receive an immediate electronic copy of the consent form, to avoid unsuitable and/or delayed randomisation. The risk of inappropriate or inadequate access to source data was mitigated in the risk assessment and data monitoring plan by stipulating approved methods of data transfer, such as using NHS.net email accounts or password-protected cloud-based systems.

#### SLiDO site survey

Table 3 shows the responses of the 20 sites represented at our 16 November 2021 site teleconference. Site representatives expressed little to no interest in continuing with either a paper site file or in-person SIV for all studies. Most of our sites had experience with the use of remote consent and felt that it had a positive effect on recruitment. The vast majority of sites felt that all of the new trial processes put into place (i.e. electronic ISF, remote SIVs, remote consent, and remote monitoring) should remain available post-COVID, but there was a clear interest in a combination of old and new methods for ISF and SIVs.

**Table 3:** Site SLiDO survey responses

	***N* (%) site respondents**
**Type of site file preferred**	(*N* = 20 sites)
Electronic file	11 (55%)
Paper file	0
Combination of electronic and paper	9 (45%)
**Preference for remote or in-person SIVs**	(*N* = 16 sites)
Remote	8 (50%)
In-person	1 (6%)
Depends on the trial	7 (44%)
**Site has consented a participant using a WILL remote consent method**	(*N* = 17 sites)
Yes	10 (59%)
If yes, do you think remote consent methods have improved recruitment at your site?	(*N* = 14 sites)
Yes	12 (86%)
No	1 (7%)
Not sure	1 (7%)
**If all COVID restrictions were lifted, remote processes sites would like to stay or to be put in place**	(*N* = 16 sites)
Having an electronic site file	14 (88%)
Remote SIVs and meetings	16 (100%)
Remote consent	16 (100%)
Remote data monitoring	13 (88%)

*SIV* Site initiation visit

## Discussion

### Summary of findings

During the UK waves of the COVID-19 pandemic, most sites reported challenges with research staffing, due to redeployment to clinical duties or to COVID-19 research. To facilitate ongoing recruitment to the WILL trial during the pandemic, adaptations were made to the investigator site file, SIVs and regular site meetings, individual-level consent, and data monitoring, all approved by the trial sponsor as pandemic adaptations, without the need for specific research ethics committee approval for implementation. Sites were almost uniformly in favour of an electronic (rather than paper) ISF; while this was not possible in order that the BCTU could comply with GCP, the online repository was established to facilitate ease of access to trial documentation. All SIVs were held remotely; this was favoured by the majority of sites, as this method offered advantages (e.g. scheduling flexibility, comprehensive team attendance, meeting recording, and reduced carbon footprint [6]), despite the disadvantages (e.g. challenges for engagement and rapport-building). Most sites used the option of remote consent, although for a minority (about one-third) of women. Sites felt that remote consenting facilitated recruitment and wished the option to be maintained. Finally, while remote data monitoring offered advantages primarily for the Central team, rather than site staff, the latter wished for this to remain.

### Comparison with literature

Our study provides a trial-specific example of COVID adaptations to clinical trial monitoring, laid out as reasonable by leads of nine CTUs in the UK Clinical Research Collaboration and Finnish Groups, during the pandemic and potentially, post-pandemic [7].

Our study addresses many of the questions posed in May 2021 to the UK CTUs regarding e-consent, defined as the use of any electronic media to convey information related to the study and to seek and/or document informed consent via an electronic device [8]. Our process mimics paper and is an easy ‘next step’ towards a fully electronic future. It was discussed with the trial sponsor and approved quickly, as a COVID adaptation that did not require REC approval before use. There was no specific IT support that had to be put into place, and sites were not required to use any one method, but could choose (and they did). Of particular note, no concerns were expressed about identifying participants correctly, or security measures (including encryption) or inappropriate use of participant signatures. A systematic review of studies of e-consent, primarily in non-interventional observational studies, suggests that e-consenting is well-received by study participants, particularly when user interfaces are readily comprehensible [9].

Our results endorse the views of 540 clinical trialists (304 from the UK and 236 from 46 other countries) who were surveyed in June/July 2020 [10]. Remote consent was viewed positively by respondents who highlighted that ‘consenting practices can be over-cautious or repetitive’, approvals can take considerable time, and resources for e-consent are inadequate within CTUs. Of note is that some of the processes that we undertook were already approved by our regulators, such as remote consent, but not used routinely; whether due to conservatism or inertia, there is no question that the pandemic encouraged innovation and risk adaption, given the new risks of COVID-19 infection for participants and staff, and the inability to complete projects in a timely fashion, if at all.

Respondents highlighted the need for flexibility and options for sites. This is a seemingly logical suggestion to deliver complex trials in diverse populations and settings where different approaches are likely to work differently. When respondents raised concerns about COVID adaptations, they focussed not on the advisability of the change, but on the resource implications. It would seem advisable to redirect at least a proportion of travel and subsistence budgets to central CTU processes, to support the ‘remote paradigm shift’, and development of CTU IT infrastructure necessary to deal with the increase in system complexity. Similar to our findings, site staff surveyed expressed mixed feelings about remote training, citing efficiency, and inclusivity as positives, but lack of in-person interaction as negatives that may affect team-building. However, they did note the potential for enhanced ongoing communication within and between teams, given the ease of joining remote meetings regularly, rather than waiting until a later date when everyone could meet in person.

### Strengths and weaknesses

Strengths of our study include a description of COVID adaptations at a reasonably large number of consultant-led maternity units in the UK, over a wide geographical distribution, within a publicly funded national healthcare service. While WILL does not involve an investigational medicinal product, it is an interventional trial and is appropriately risk-adapted.

Limitations include direct survey responses from a subset of half of active WILL trial sites. Our processes may only work as well if Central team members are able to work from a private environment (such as home), rather than from open, shared spaces where videoconferencing is not always possible and room bookings are at a premium.

## Conclusions

To facilitate ongoing recruitment to the WILL trial during the COVID-19 pandemic, adaptations were made to the WILL protocol and trial procedures that facilitated fully remote conduct, and were implemented rapidly, with sponsor approval, but without the need for research ethics committee approval. While there were pros and cons of new processes, Central team members and site trial staff were enthusiastic about having an electronic ISF and maintaining the option of remote SIVs and videoconferencing, remote consent, and remote data monitoring. COVID created a choice to adapt the trial to the local context.

Remote trial processes should be included in future trial design and funding applications. These processes empower recruits with the choice about consent and participation wherever possible. This would be a positive research legacy of COVID-19.

## Data Availability

The datasets used and/or analysed during the current study are available from the corresponding author on reasonable request.

## Abbreviations

BCTU: Birmingham Clinical Trials Unit
BP: Blood pressure
CI: Chief investigator
COVID-19: SARS-CoV-2
CTU: Clinical trials unit
eISF: Electronic investigator site file
GCP: Good clinical practice
HRA: Health Research Authority
ISF: Investigator site file
IT: Information technology
MHRA: Medicines and Healthcare products Regulatory Agency
NHS: National Health Service
NIHR: National Institute for Health Research
PI: Principal investigator
SIV: Site initiation visit
TMF: Trial master file
UK: United Kingdom
WILL: When to Induce Labour to Limit risk in pregnancy hypertension
